# Pre-purchase screening for *Coxiella burnetii* in small ruminants: farm acceptance and field evaluation identify the *ex-vivo* interferon-γ assay as a promising tool

**DOI:** 10.3389/fvets.2025.1708200

**Published:** 2025-12-04

**Authors:** Anneleen Matthijs, François Claine, Xavier Simons, Ana Soares, Damien Desqueper, Eva Van Mael, Marcella Mori

**Affiliations:** 1 Sciensano, Belgian Institute for Health, Brussels, Belgium; 2 National Reference Centre for Coxiella burnetii and Bartonella, Brussels, Belgium; 3 Association Régionale de Santé et d'Identification Animales (Arsia), Ciney, Belgium; 4 Dierengezondheidszorg Vlaanderen (DGZ), Torhout, Belgium

**Keywords:** *Coxiella burnetii*, small ruminants, survey, IFN-γ recall assay, ELISA, real-time qPCR, intradermal test

## Abstract

**Introduction:**

Q fever, caused by *Coxiella burnetii*, is a disease posing significant zoonotic risk and economic impact, particularly in small ruminants. Screening prior to flock introduction is essential for disease control and prevention, especially in breeding males, given their potential role in pathogen transmission.

**Methods:**

This study combined a survey of small ruminant farmers with a field evaluation to explore the feasibility of pre-purchase screening and to compare the performance of four diagnostic tests: real-time qPCR, antibody ELISA, intradermal testing, and an *ex-vivo* interferon-gamma (IFN-γ) recall assay.

**Results:**

The survey (*n* = 114) revealed widespread gaps in health status awareness and a lack of pre-screening during animal lending or borrowing, with regional differences, although most farmers supported pre-purchase screening. The field evaluation across ten farms (five positive and five negative) showed that the IFN-γ recall assay provided diagnostic accuracy comparable to ELISA (DSe: 0.80 vs. 0.74; DSp: 0.94 vs. 0.96), with higher positivity rates among unvaccinated animals (*n* = 122; 42.6% vs. 26.2%). The assay also proved feasible for field use, with a 24-hour antigen stimulation protocol performing equally good as the 48-hour version (AUC: 0.99 vs. 0.98). Real-time qPCR and intradermal testing showed the least utility under field conditions.

**Discussion:**

Overall, the IFN-γ recall assay emerges as a promising diagnostic tool for detecting *C. burnetii* infection in small ruminants, particularly in settings where vaccination history is unknown or incomplete.

## Introduction

Q fever is a zoonotic disease caused by the intracellular bacterium *Coxiella burnetii*. In Belgium, it is a notifiable infection in animals and humans and subject to a national surveillance program aimed at reducing the risk of its spread from animals (Law of 24 March 1987 on animal health, Royal Decree of 3 February 2014, and Ministerial Decree of 11 May 2011). Small ruminants are the main source of infection in humans (1, 2). Infected females can release large quantities of the *C. burnetii* into the environment during abortion or normal parturition, primarily through birth products. The bacterium is also shed in milk, feces, and urine (3–8). Since 2011, Belgium has implemented official monitoring programs in animals focused on detecting the pathogen in abortion products of sheep and goats, and on the regular screening of bulk tank milk in small ruminant dairy farms (9, 10). As infection in naïve herds represents a danger to public and animal health, identification of a positive herd results in a series of necessary and severe biosecurity measures that are legally imposed on the affected farm. In addition, vaccination is enforced to the entire flock—which is a costly endeavor for the farmers. It is, therefore, important to develop and promote actions to help the farmer minimize the risk of disease introduction into the herd.

In low-endemicity settings, acquisition of new animals is a proven risk factor for herd infection (11). Few studies have attempted to demonstrate this risk when purchasing untested breeding bucks and rams, particularly knowing that *C. burnetii* can be sexually transmitted, as demonstrated by a study in mice (12). The bacterium has also been detected in the semen of bulls and rams (13, 14) and, more recently, in the prepuce of rams (11), suggesting that *C. burnetii* transmission can occur during sexual intercourse of small ruminants.

Within the current Belgian monitoring programs, screening of breeding males for *C. burnetii* infection before their introduction into the flock is not performed. This omission increases the risk of potential infection in the females they service and could cause rapid amplification of infection within the herd. Testing breeding bucks and rams before introduction supports strategies to reduce the risk of introduction and spread of *C. burnetii* within herds. Moreover, males can also serve as effective sentinel animals, as they come into close contact with multiple females in the flock during the breeding period, and sampling of those animals could help to monitor the herd’s sanitary status.

In Belgium, two types of individual *C. burnetii* screening tests in live animals are routinely available for ruminants: an indirect ELISA, which detects specific humoral immune response in serum or milk, and a real-time qPCR assay, which can be performed on genital swabs, feces, and milk. The *C. burnetii*-specific antibody ELISA is an indirect tool detecting exposure, while PCR testing allows direct detection of the pathogen and thus identification of active infections. However, some infected animals may be missed through PCR testing due to intermitted shedding of the bacteria in vaginal mucus, feces, and milk (15, 16). Also, with ELISA, the number of false negatives can be high—as up to 25% (8)—as not all infected animals develop a detectable antibody response (8, 15, 17). Moreover, antibody ELISA testing cannot discriminate between vaccinated and naturally exposed animals. As *C. burnetii* is an intracellular bacterium, cell-mediated immunity (CMI) testing might tackle some of the above shortcomings and offer an interesting diagnostic alternative. A *C. burnetii*-specific interferon-γ (IFN-γ) recall assay—involving (i) *ex-vivo* peripheral blood mononuclear cells (PBMCs), (ii) *C. burnetii in-vitro* antigen stimulation (varying from overnight to 48 h) and (iii) subsequent IFN-γ detection in the supernatant—was successfully deployed in experimental Q fever research settings (18, 19). This approach enabled the early detection of infection in non-vaccinated, challenged animals, suggesting superior sensitivity compared to serum antibody ELISA and owing that an IFN-γ recall assay holds significant potential as a field diagnostic tool (18). A second test that measures CMI is the intradermal test, where the inactivated antigens are injected into the skin, and the delayed-type hypersensitivity reaction is measured by the size of the induration. In human medicine, this test is used to evaluate the immune status of patients before vaccination with the Q fever vaccine Q-VAX® (CSL Ltd., Victoria, Australia), to avoid unwanted secondary vaccine reactions. A French research group investigated the use of the skin test method in cattle to detect *C. burnetii*-immune animals within a herd and found it to be an easy, low-cost tool to determine the subpopulation of cows to be (re)vaccinated (20, 21). This test has not yet been appraised in small ruminants under field settings.

To date, no large-scale study has been conducted in Belgium to evaluate the pertinence of (pre-purchase) screening of individual animals—particularly breeding bucks and rams—for preventing *C. burnetii* (re-)infections, nor have different *C. burnetii*-specific CMI tests been evaluated and compared for their applicability in small ruminants under field conditions.

In this study, by means of a questionnaire addressed to the Belgian sheep and goat farmers, we initially assessed the farmers’ acceptance to adopt a (pre-purchase) screening test for Q fever in breeding bucks and rams. Secondly, we evaluated the diagnostic performance of two *C. burnetii*-specific CMI tests for sheep and goats under field circumstances, i.e., the *in-vivo* intradermal test and the *in-vitro* IFN-γ recall assay (24-h vs. 48-h antigen stimulation) on whole blood. These CMI tests were finally compared for their diagnostic potential with that of established methods, such as *C. burnetii* real-time qPCR on genital swabs and *C. burnetii*-specific antibody ELISA testing on serum.

## Materials and methods

### Design and distribution of the survey to the goat and sheep farmers

To gain deeper insight into the management of breeding males on sheep and goat farms, and to assess the farmers’ acceptance of a screening test for Q fever and other sexually transmitted diseases, an online survey was developed in Dutch and French using Google Forms. The questionnaire comprised 43 questions addressing: general farm characteristics (*n* = 7); breeding management (*n* = 7); and management of the breeding males in particular (*n* = 19); animal health (*n* = 3); and perception regarding a pre-purchase screening test for Q fever and other sexually transmitted diseases (*n* = 5). In the last two questions, the farmer was inquired about his interest to be enrolled in the field study of this project. The full version of the questionnaire (in French and Dutch) is provided in the Supplementary Materials. The link to the online questionnaire was distributed by e-mail among the small ruminant farmers in Belgium by Dierengezondheidszorg Vlaanderen (DGZ) and Association Régionale de Santé et d’Identification Animales (ARSIA) in December 2021. The survey was closed in March 2022.

### Farm selection and sampling strategy

Considering results of the questionnaire and information from the national Q fever surveillance programs, five Q fever-positive and five Q fever-negative small ruminant farms were recruited throughout Belgium. A farm was considered positive when one or more abortion cases tested PCR-positive for *C. burnetii* or when the bacterium was detected by PCR in bulk tank milk in the past year. Farms were considered as negative if they had never obtained a *C. burnetii*-positive PCR or positive antibody ELISA result in the abortion protocol or bulk tank milk screening. An overview of the participants and their main farm characteristics is shown in Table 1.

**Table 1:** Overview of the participating farms and their main characteristics.

Farm	Q fever status	Q fever vaccination	Region	Type + activity	N females of reproductive age	N breeding males
1	Positive	No	Wallonia	Ovine Meat	15	2
2	Positive	No	Flanders	Mixed Milk	21 ewes	1 ram
					13 does	2 bucks
3	Positive	No	Flanders	Mixed Meat/repro	±70 ewes	2 rams
					±50 does	2 bucks
4	Positive	Yes (males not)	Wallonia	Caprine Milk	±150	4
5	Positive	Yes	Wallonia	Ovine Milk	±40	3
6	Negative	No	Wallonia	Ovine Repro	8	1
7	Negative	No	Wallonia	Caprine Milk/repro	±40	1
8	Negative	No	Wallonia	Ovine Meat/repro	±70	3
9	Negative	No	Flanders	Caprine Milk/repro	±50	1
10	Negative	No	Flanders	Caprine Milk/repro	±180	3

**Figure 1:**
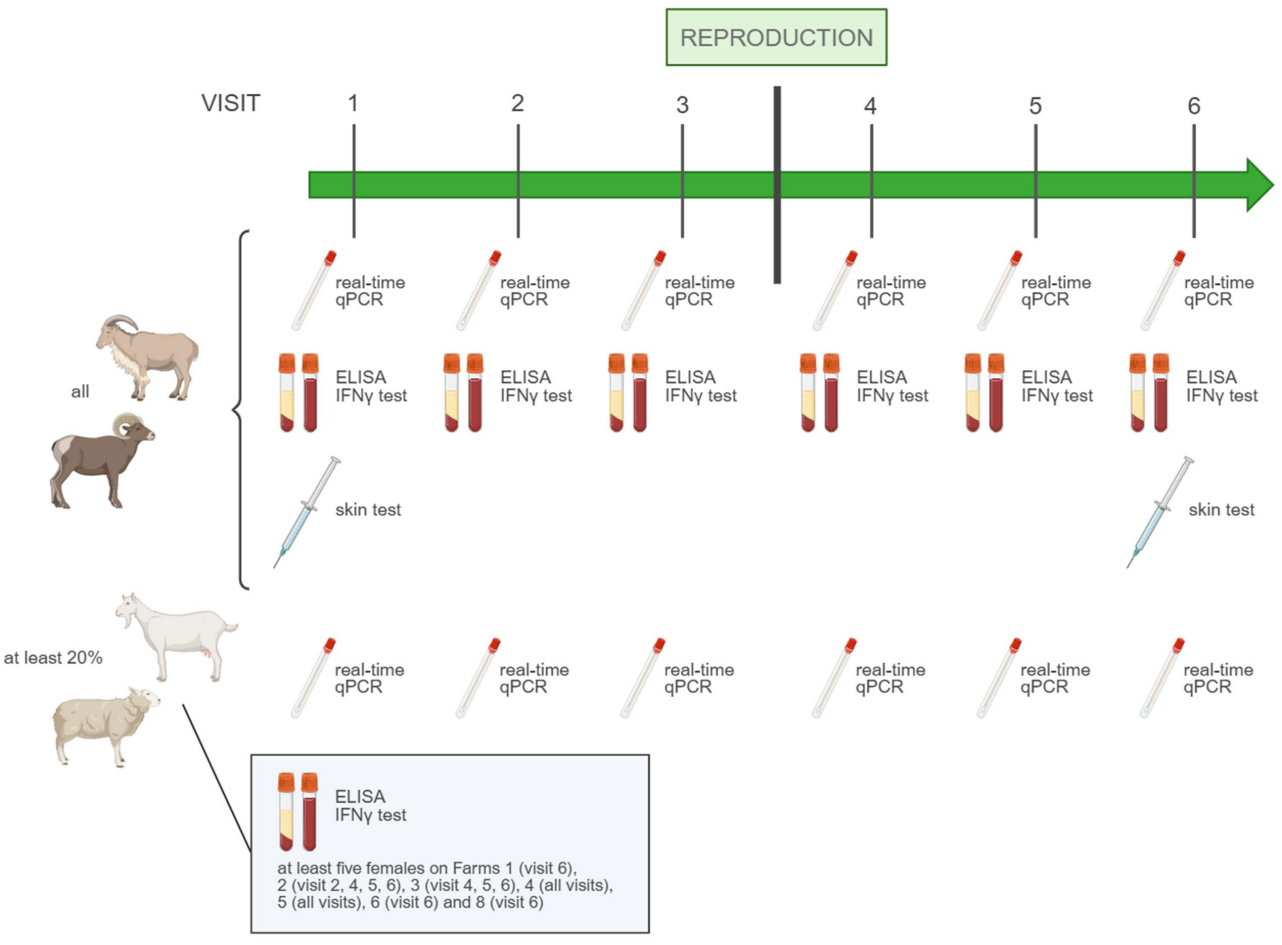
Sampling protocol of the field study to evaluate four types of Q fever screening tests.

Between May 2022 and February 2024, six farm visits were attempted on each farm, three before and three after breeding, with an interval of roughly 1 month between visits. During each visit, dry preputial swabs were collected from all breeding males for *C. burnetii* real-time qPCR, as well as serum on BD Vacutainer® SST™ Advance tubes (BD Bioscience, San Jose, CA, United States) and whole blood samples on BD Vacutainer® LH 170 I.U. tubes (BD Bioscience, San Jose, CA, United States) for *C. burnetii* antibody ELISA and the IFN-γ recall assay, respectively. In addition, the *C. burnetii* infection status of at least 20% of the females in reproductive age was monitored on each farm—and per species on mixed farms—by dry vaginal swabbing and subsequent *C. burnetii* real-time qPCR. If the number of females in reproductive age was smaller than 30 all females were sampled. Additional serum and heparinized whole blood samples for *C. burnetii* antibody ELISA and the *C. burnetii*-specific IFN-γ recall assay, respectively, were collected of at least five females (per species on mixed farms) on Farms 1 (visit 6), 2 (visit 2, 4, 5, 6), 3 (visit 4, 5, 6), 4 (all visits), 5 (all visits), 6 (visit 6), and 8 (visit 6). On the first and last visits, the breeding males underwent intradermal testing (Figure 1).

### DNA extraction and *C. burnetii* real-time qPCR

Preputial and vaginal swabs were placed into microtubes containing 1 mL of sterile physiological saline and thoroughly vortexed. DNA was extracted from 200 μL of liquid using the MagMAX™ Total Nucleic Acid Isolation kit (Applied Biosystems™ Thermo Fisher Scientific Inc., Waltham, MA, United States) according to the manufacturer’s instructions. Next, a real-time qPCR targeting the IS1111 repetitive element of *C. burnetii* and ruminant beta-actin (used as an internal positive control) was performed as previously described by Mori et al. (10), using either the 7,500 Real-Time PCR System (Applied Biosystems™ Thermo Fisher Scientific Inc., Waltham, MA, United States) or LightCycler® 480 Instrument II (Roche Molecular Systems Inc., Pleasanton, CA, United States). Results were expressed as cycle threshold (C*t*) values. Samples with a C*t* value below 40 for *C. burnetii* were considered positive, regardless of the C*t* value for the beta-actin control, while C*t* values equal to or greater than 40 for *Coxiella* and below 40 for beta-actin were deemed negative. Samples with a C*t* value equal to or greater than 40 for both *C. burnetii* and beta-actin were classified as inhibited and excluded from data analysis.

### Antibody testing

Serum samples were analyzed within the laboratory’s routine accredited workflow for the presence of antibodies against *C. burnetii* with a commercial indirect *C. burnetii* antibody ELISA (PrioCHECK™ Ruminant Q Fever Ab Plate Kit, Applied Biosystems™ Thermo Fisher Scientific Inc., Waltham, MA, United States) according to the manufacturer’s instructions. Samples with a sample-to-positive control ratio (S/P%) bigger than 40 were classified as positive. Samples with an S/P% equal or smaller than 40 were considered negative.

### Intradermal testing

The breeding males underwent intradermal testing by intradermal injection of 0.1 mL of a 1/3 dilution of Coxevac® (Ceva Santé Animale, Libourne, France) in physiological saline applied in the neck region after clipping. The skin reaction was evaluated by the farmer after 72 h to up to a week after injection using a scoring system ranging from 0 to 4 (22). The score considered the presence and diameter of skin thickening at the injection site. A score of 1 (“obvious thickening of the dermis: nodule between 1 and 1.5 cm in diameter approximately”) or higher was considered as a positive test result. In addition, when possible, the lecture was documented through photographs and re-evaluated by the investigating scientists.

### Interferon-γ recall assay

Briefly, 900 μL of heparinized whole blood was incubated in duplicate with 100 μL of heat-inactivated *C. burnetii* antigens derived from the Belgian field strain CbBEB2, 100 μL of pokeweed mitogen (PWM) at 5 μg/mL (positive control), and 100 μL of phosphate-buffered saline (PBS; negative control) in Thermo Scientific™ Nunc™ sterile, non-treated 24-well plates (Thermo Fisher Scientific Inc., Waltham, MA, United States) at 37 ± 2 °C. After 24 h, 100 μL of plasma was harvested following centrifugation (10 min at 500–700 g) and stored at −20 °C. The pellet was resuspended, and plates were incubated for another 24 h at 37 ± 2 °C. Afterward, the plates were centrifuged, and all plasma was collected and stored at −20 °C. Interferon-γ was detected in the plasma harvested after 24-h and 48-h stimulation using the commercial sandwich ELISA ID Screen® Ruminant IFN-γ (IDVet, Montpellier, France) according to the manufacturer’s instructions. Results were expressed as S/P% and, for each stimulation, the mean of the two duplicate values was used for further analysis. For each tested sample, the test was considered valid if S/P% was higher than 50 for the positive (PWM) control, and below 50 for the negative (PBS) control.

### Statistical analyses

To evaluate the ability of the IFN-γ recall assay to discriminate between *C. burnetii*-infected and naïve animals and to compare the performance of two plasma collection timepoints (24 h and 48 h), a receiver operating characteristics (ROC) analysis was performed in GraphPad Prism software version 9.4.1 (San Diego, CA, United States) for both conditions. The analysis was performed using S/P% test results from both *C. burnetti* real-time qPCR- and serologically negative animals originating from Q fever-negative farms (“naïve”; *n* = 42), as well as *C. burnetii* real-time qPCR-positive and/or serologically positive animals from the non-vaccinating Q fever-positive Farm 2 (“infected”; *n* = 30). The ROC curves were generated by plotting the true positive rate [test sensitivity (Se)] against the false positive rate (1-test specificity (Sp)) against various thresholds, allowing the selection of optimal test thresholds for positivity. Cohen’s Kappa was used to quantify the level of agreement between tests in IBM® SPSS® Statistics version 30.0.0.0 (Armonk, NY, United States). Differences in result proportions between tests were assessed by McNemar’s test in IBM® SPSS® Statistics version 30.0.0.0 (Armonk, NY, United States), and differences in quantitative test results between conditions were analyzed using Mann–Whitney U tests after confirmation of non-normal distribution with the Kolmogorov–Smirnov test in GraphPad Prism software version 9.4.1 (San Diego, CA, United States).

As no gold standard exists to unambiguously determine the positive/negative status of an animal, a hierarchical latent class model (LCM) was used to estimate the diagnostic sensitivity (DSe) and diagnostic specificity (DSp) of the IFN-γ and ELISA tests, as proposed previously by Hanson et al. (23). As Q fever prevalence can be different in each herd, the model assumes that a distribution of disease prevalence exists across herds. Tests are assumed to be independent, and their DSe and DSp are considered constant within the studied population. Non-informative Beta (1.0, 1.0) distributions were used as priors for DSe and DSp of both tests. The model code is provided in Supplementary Script 1 and Supplementary Table 1.

The Bayesian analysis was performed using the Just Another Gibbs Sampler (JAGS) software and processed with the R2jags and coda packages in R (24, 25). The inferences presented were based on Markov chain Monte Carlo (MCMC) samples comprising 75,000 iterations, constituting of three different chains of 25,000 iterations with different initial values, obtained after a burn-in period of 5,000 samples. Visual inspection of trace plots was used to check chains’ convergence.

## Results

### Evaluation of the acceptance and need of a (pre-purchase) screening test for Q fever in breeding bucks and rams

A total of 114 farmers filled in the questionnaire: 52 (45.6%) from Wallonia and 62 (54.4%) from Flanders, representing all the 10 Belgian provinces. The majority of the participants were meat-producing sheep farmers (Supplementary Figure 1). The mean herd size of the Walloon farms was 78 heads (min: 6 and max: 380) and 250 heads (min: 5 and max: 2000) in Flanders.

Questioning on the origin of breeding males revealed that more than half of the small ruminant farmers in both Wallonia (59.6%) and Flanders (53.2%) relied exclusively on purchased breeding males. A smaller proportion of farmers (38.4% in Wallonia and 37% in Flanders) combined purchased males with those that are borrowed and/or born on their own farm. Exclusively borrowing males for breeding was rare in both regions (1.9% in Wallonia and 1.6% in Flanders), whereas the sole use of home-born animals was reported only by Flemish farmers (8.1%). Additionally, 28.8% of Walloon and 17.7% of Flemish farmers lent breeding males to other farms. Interestingly, almost half of the Walloon farmers and one-fourth of the Flemish farmers were unaware of the health status of the farm(s) of origin when purchasing and/or borrowing breeding males. Approximately one-third of the Flemish farmers and up to two-thirds of the Walloon farmers were unaware of the vaccination status of the breeding males that they purchased and/or borrowed, and the majority of the farmers did not screen these animals for infectious diseases before introducing them into their herd. When breeding males were lent to other farms, similar numbers were observed regarding the unawareness of the health status of the farm(s) to which animals are lent, and the lack of screening of lent animals before re-introduction into their proper herd (Table 2). The questionnaire also revealed that 25.8% of the farmers in Flanders did not vaccinate their breeding males when vaccinating against diseases, while in Wallonia this percentage was twice as high (Table 2). When questioned on the usefulness of systematically screening breeding males for sexually transmitted diseases upon purchase, 61.3% of the Flemish farmers and up to 78.8% of the Walloon farmers considered testing pertinent, with “maintaining a disease-free status” as main motivation to use such a test. However, slightly more than half of the farmers indicated that the cost price of such a test would be the main limiting factor for use. Approximately one-third of the farmers defined other reasons for not using such a test. Among those “a lack of knowledge of the diseases and available tests” and “purchases from high health farms only” were the most cited ones. The anonymized responses corresponding to the questions discussed above and presented in Table 2 are provided in the Supplementary Materials.

**Table 2:** Management of the breeding males on Walloon and Flemish sheep and goat farms, and perception of the small ruminant farmers regarding a screening test for sexually transmitted diseases at purchase.

Origin of the breeding males.		Walloon farmers (%)		Flemish farmers (%)	
The breeding males are…					
	purchased	59.6		53.2	
	purchased, borrowed	3.8		3.2	
	purchased, born on your farm	23.1		29.0	
	purchased, borrowed, born on your farm	11.5		4.8	
	borrowed	1.9		1.6	
	born on your farm	0.0		8.1	
Percentage of farmers that lends breeding males to other farms.		28.8		17.7	
If breeding males are purchased and/or borrowed:		*Yes*	*No*	*Yes*	*No*
Are you aware of the health status of all farms of origin?		52.0	48.0	73.7	26.3
Do you know the vaccination status of all animals purchased and/or borrowed?		34.0	66.0	71.9	28.1
Are purchased and/or borrowed animals screened for certain infectious diseases before being introduced into the herd?		20.0	80.0	31.6	68.4
If breeding males are lent to other farms:		*Yes*	*No*	*Yes*	*No*
Are you aware of the health status of all farms to which animals are lent?		40.0	60.0	81.8	18.2
Are lent animals screened for certain infectious diseases before being re-introduced into the herd?		0.0	100.0	36.4	63.6
General management of the breeding males.		*Yes*	*No*	*Yes*	*No*
If you vaccinate against diseases, are the breeding males vaccinated as well?		44.2	55.8	74.2	25.8
How are the ewes/does serviced?					
	Exclusively by the ram/buck (natural service)	100.0		90.3	
	By the ram/buck (natural service) and by artificial insemination	0.0		9.7	
	By artificial insemination only	0.0		0.0	
Farmers’ perception of a pre-purchase screening test for sexually transmitted diseases.		*Yes*	*No*	*Yes*	*No*
Do you think it is useful to systematically screen breeding males for sexually transmitted diseases (e.g., leptospirosis, chlamydiosis, Q fever) at the time of purchase?		78.8	21.2	61.3	38.7
What is your main motivation for using such a test?					
	Obtain more information on the health status of purchased animals	32.7		19.4	
	Improve health status of the herd by reducing infection pressure	25.0		22.6	
	Maintain a disease-free herd status	42.3		53.2	
	Other	0.0		4.8	
What is your main motivation for NOT using such a test?					
	The cost price	59.6		56.5	
	Uncertainty about the continuation of the sale	11.5		4.8	
	Other	28.9		38.7	

### Field evaluation of four *C. burnetii* screening tests

Considering the high number of farmers unaware of or not screening the health status of breeding males before (re)introduction into the herd, we evaluated the field pertinence of four Q fever screening tests, namely real-time qPCR, antibody ELISA, intradermal testing and an *ex-vivo* IFN-γ-based blood recall assay in five positive and five negative farms. Farms 2, 9, and 10 were enrolled near the beginning of the breeding season. As a result, only two, one, and none out of three pre-breeding visits were conducted on Farm 2, 9, and 10, respectively. For Farm 10, follow-up of a second breeding batch was attempted in the following year, which allowed for the completion of three pre-breeding visits. However, after the third visit, the farmer decided not to proceed with reproduction due to the cessation of milking activities, and consequently, the three post-breeding visits could not be conducted in the second year. Similarly, on Farm 9, the final post-breeding visit was not conducted due to the sale of animals. Interim results showed low positivity rates among the males in all the four tests. Therefore, it was decided to collect blood samples from the females as well for antibody ELISA and IFN-γ testing, more frequently on Q fever-positive farms and less frequently on Q fever-negative farms, to increase the likelihood of obtaining positive test results. At the time this decision was made, sampling had already been completed or was nearly completed on some farms, while on others it had not yet started or just begun, resulting in inconsistent blood sampling of the females across the farms. In total, 244 animals (27 males and 217 females) were sampled during the whole study period, resulting in the analysis of 1,223 genital swabs, 253 whole blood samples, and 254 serum samples. The intradermal test was performed 44 times.

#### Detection rates with real-time qPCR

Of the 1,223 genital swabs, 1,221 were considered eligible for further analysis, as two showed PCR inhibition. On Q fever-positive farms, only 7 out of 777 swabs (0.9%) collected from 149 animals tested positive. Five of these positive swabs came from four ewes on Farm 2, while the remaining two were from two does on Farm 4. The bacterium was not detected in any swabs collected from the Q fever-negative farms (Table 3).

**Table 3:** The number (percentage) of genital swabs and serum samples that tested positive in the *C. burnetii* real-time qPCR and *C. burnetii*-specific antibody ELISA, respectively, as well as the number (percentage) of whole blood samples testing positive or doubtful in the *C. burnetii*-specific IFN-γ recall essay for both Q fever positive and negative farms.

Farms	Pre-breeding		Post-breeding		Total	
*C. burnetii* real-time qPCR						
	N positive (%)		N positive (%)		N positive (%)	
Q fever-positive	6/389 (1.5)		1/388 (0.3)		7/777 (0.9)	
Q fever-negative	0/214 (0.0)		0/230 (0.0)		0/444 (0.0)	
Total	6/603 (1.0)		1/618 (0.2)		7/1221 (0.6)	
*C. burnetii* antibody ELISA						
	N positive (%)		N positive (%)		N positive (%)	
Q fever-positive	44/75 (58.7)		40/121 (33.1)		84/196 (42.9)	
Q fever-negative	5/24 (20.8)		3/34 (8.8)		8/58 (13.8)	
Total	49/99 (49.5)		43/155 (27.7)		92/254 (36.2)	
*C. burnetii* IFN-γ recall assay						
	N positive (%)	N doubtful (%)	N positive (%)	N doubtful (%)	N positive (%)	N doubtful (%)
Q fever-positive	43/74 (58.1)	5/74 (6.8)	55/121 (45.5)	15/121 (12.4)	98/195 (50.3)	20/195 (10.3)
Q fever-negative	4/20 (20.0)	2/20 (10.0)	0/34 (0.0)	1/34 (2.9)	4/54 (7.4)	3/54 (5.6)
Total	47/94 (50.0)	7/94 (7.4)	55/155 (35.5)	16/155 (10.3)	102/249 (41.0)	23/249 (9.2)

#### Detection rates with antibody testing

On Q fever-positive farms, 57 animals were sampled, yielding a total of 196 serum samples. ELISA detected antibodies in 84 of these samples (42.9%), all originating from Farms 2 through 5. In total, 25 animals (seven males and 18 females) had at least one positive test result. On Farm 1, none of the sampled animals had detectable antibody levels. On Q fever-negative farms, 8 out of 58 serum samples (13.8%) collected from 21 animals tested positive. All positive samples originated from Farm 10, where one buck consistently tested positive across the six sampling points. Additionally, another breeding buck seroconverted during the pre-breeding visits of year 2, yielding the other two positive samples.

#### Detection rates with intradermal testing

Of the 44 intradermal tests conducted on 27 males, only one yielded a positive result, which was observed in an unvaccinated breeding buck from Farm 4.

#### Detection rates with IFN-γ recall assay

**Figure 2:**
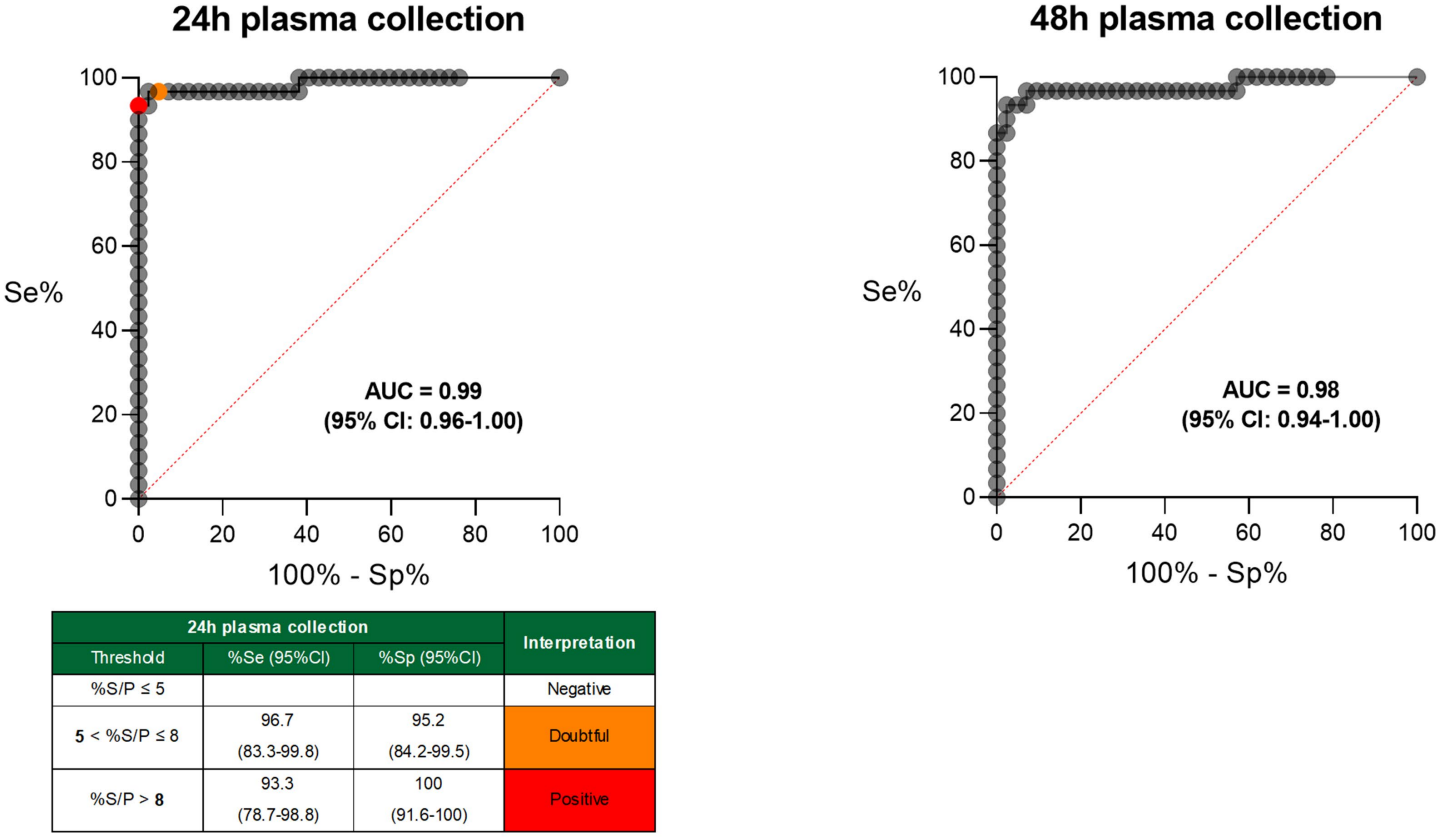
Receiver operating characteristics (ROC) curves for both plasma collection time points (after 24-h and 48-h antigen stimulation) of the *C. burnetii*-specific IFN-γ recall assay. AUC, area under the curve; CI, confidence interval; Se, sensitivity; Sp, specificity; %S/P, sample-to-positive control ratio.

Receiver operating characteristics (ROC) analyses performed with S/P% results after 24-h and 48-h stimulation from naïve and infected animals allowed the evaluation of the discriminatory ability of the IFN-γ recall assay itself, comparison of both plasma collection timepoints and determination of the threshold for positivity. The area under the curve (AUC) was 0.99 (95% CI: 0.96–1.00) and 0.98 (95% CI: 0.94–1.00) for the 24-h and 48-h stimulation, respectively, showing a comparable and excellent discriminatory ability of both plasma collection timepoints. As shorter test turnaround times are more suitable for field testing, only the results from the 24-h plasma collection timepoint were considered for further analysis. For this timepoint, a threshold of >8 S/P% was established to classify samples as positive, ensuring 100% test Sp while maximizing test Se (93;3%). To increase Se to 96.7%, a second, lower threshold was identified, allowing results within this range to be classified as “doubtful” (Figure 2).

Of the total 253 whole blood samples analyzed, four had invalid test results (S/P% of positive PWM control < 50) and were excluded from further analysis (Table 3). On Q fever-positive farms, 98 out of 195 samples (50.3%) collected from 57 resident animals tested positive, with positive results found across all five farms. Thirty-five different animals (seven males and 28 females) had at least one positive result. On the Q fever-negative farms, a total of 21 animals were followed, resulting in 54 samples. Four of these (7.4%) tested positive, originating from two breeding bucks on Farm 10 in year 2 (i.e., one who seroconverted at the same time and one who remained seronegative during the entire study).

Twenty out of 195 samples (10.3%) were classified as doubtful on the Q fever-positive farms, originating from 14 different animals. Eleven from these animals (yielding 15/20 doubtful results) had also at least one positive IFN-γ test result during the study. On the Q fever-negative farms, the number of doubtful results was twice as less (3/54; 5.6%), yielding from two breeding males on Farm 7, and the breeding buck on Farm 10 that tested seropositive during the entire study. In further analyses, doubtful results were considered negative.

#### Comparison of the diagnostic performance of the four *C. burnetii* screening tests

Given that only a few positive results were obtained using PCR and intradermal tests, and that data were not available for all time points, it was only possible to compare the diagnostic performance of ELISA and IFN-γ tests. In total, 247 samples from the 10 herds were available for these two tests, originating from 78 animals. The few PCR- and skin test-positive results were used to interpret and confirm the ELISA and IFN-γ test results (Table 4). Cohen’s kappa indicated that the agreement between the ELISA and IFN-γ tests was moderate to strong (*κ* = 0.58; 95% CI: 0.47–0.68). DSe and DSp of the IFN-γ recall assay and ELISA were assessed using a hierarchical Bayesian LCM. The analysis showed that both tests performed on a similar level (DSe of 0.80 (95% CI: 0.69–0.92) and DSp of 0.94 (95% CI: 0.88–0.996) for the IFN-γ recall assay vs. DSe of 0.74 (95% CI: 0.63–0.84) and DSp of 0.96 (95% CI: 0.89–0.998) for ELISA), with the IFN-γ test being slightly less specific and a bit more sensitive than the ELISA.

**Table 4:** Summary of *C. burnetii* ELISA, IFN-γ recall assay, real-time qPCR, and intradermal test results by farm.

Farm			ELISA/IFN-γ				N samples	Positivity rate (%)		Positivity in other tests	
ID	Q fever status	Vaccination	E+/I+	E+/I-	E-/I+	E-/I-		E	I	N + PCR	N + skin test
1	Pos	No	0	0	1	15	16	0	6	0	0
2	Pos	No	24	1	12	5	42	60	86	5	0
3	Pos	No	3	2	6	38	49	10	18	0	0
4	Pos	Yes^*^	29	4	4	9	46	72	72	2	1
5	Pos	Yes	14	7	5	14	40	53	48	0	0
6	Neg	No	0	0	0	9	9	0	0	0	0
7	Neg	No	0	0	0	6	6	0	0	0	0
8	Neg	No	0	0	0	18	18	0	0	0	0
9	Neg	No	0	0	0	3	3	0	0	0	0
10	Neg	No	2	6	2	8	18	44	22	0	0
Total			72	20	30	125	247			7	1

E, ELISA; I, IFN-γ; +, positive result; −, negative result.

* The three males of Farm 4 were not vaccinated.

**Figure 3:**
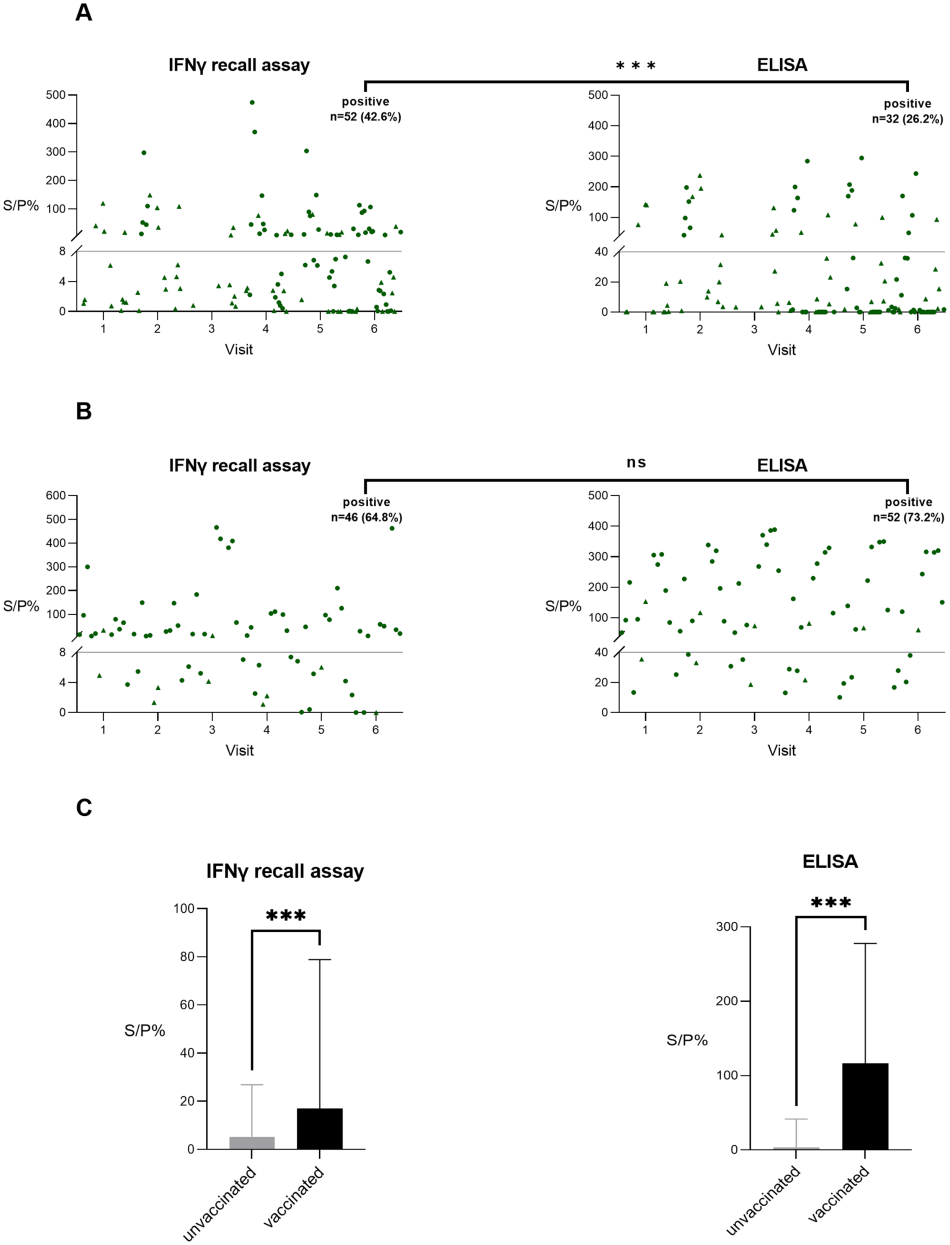
*Coxiella burnetii* IFN-γ recall essay and *C. burnetii* antibody ELISA test results originating from both Q fever-unvaccinated and vaccinated animals residing on Q fever-positive farms. **(A)** Samples (*n* = 122) originating from unvaccinated animals and **(B)** samples (*n* = 71) originating form vaccinated animals. Samples above the cut-off line (full grey line) are considered positive, samples situated below the cut-off are negative. Differences in the number of positive test results between tests were assessed by McNemar’s test. (▲) sample from male. (●) sample from female. **(C)** Median and interquartile range of quantitative test results from vaccinated and unvaccinated animals were compared using the Mann–Whitney *U*-test. ns, non-significant; *p < 0.05; **p < 0.01; ***p < 0.001; S/P%, sample-to-positive control ratio.

The ELISA and IFN-γ positivity rates in positive farms ranged from 0 to 86%. Interestingly, when stratifying the Q fever test results into two groups—unvaccinated and vaccinated animals from positive farms—the IFN-γ recall assay yielded significantly more positive results than the ELISA in the unvaccinated group (42.6% (52/122 samples) for IFN-γ test vs. 26.2% (32/122 samples) for ELISA, *p* < 0.001, Figure 3A). In contrast, among vaccinated animals, no significant difference was observed between the two methods (64.8% (46/71 samples) in IFN-γ test vs. 73.2% (52/71 samples) in ELISA, *p* > 0.05, Figure 3B). This indicated a higher sensitivity of the IFN-γ recall assay in absence of vaccination. To further investigate to which extent vaccination interferes with the results of both tests, quantitative (S/P%) results from vaccinated and unvaccinated animals were compared. For both the IFN-γ recall assay as the ELISA, the median of the results originating from vaccinated animals was significantly higher than those from unvaccinated ones (Figure 3C). However, the difference between the two conditions was more pronounced for the ELISA (33-fold difference between medians) compared to the IFN-γ recall assay (three-fold difference between medians). These results indicate that the antibody ELISA is affected the most by vaccine-induced responses, leading to pronounced differences in antibody titers.

Of the 78 animals tested with the ELISA and IFN-γ recall assay, four animals (Farm 2) had positive PCR results. Three ewes were PCR-positive at the first visit before breeding. While these animals were not tested with the ELISA and IFN-γ recall assay at that timepoint, they tested positive at least once in both assays during subsequent visits. One ewe was PCR-positive at visit 1 before breeding and at visit 1 after breeding. This ewe was blood-sampled at the second visit before breeding and the two first visits after breeding, and tested positive in both the ELISA and IFN-γ tests at all three timepoints. No blood samples were collected from the two PCR-positive animals on Farm 4 during the study period. Intradermal skin tests were conducted on a limited subset of 27 males at only two timepoints: the first visit before reproduction and the last visit after reproduction. Of the 44 samples analyzed, only one positive result was observed on Farm 4 (last visit after reproduction). At this timepoint, the buck tested positive by IFN-γ, but negative by ELISA. He had tested IFN-γ-positive during all five previous visits, and ELISA-positive at visits 2 and 3 before breeding.

## Discussion

In this study, we first sought to assess the applicability and acceptance of a (pre-purchase) screening test for Q fever in breeding bucks and rams, using a questionnaire addressed to Belgian sheep and goat farmers. At the time of dissemination of the questionnaire (2021), the Belgian small ruminant population counted 269,843 sheep and 124,903 goats, distributed across 27,165 and 12,018 herds, respectively. While sheep are primarily kept for meat production, wool, natural grazing, or hobby purposes, and to a lesser extend for milk, goats are mainly kept for dairy production, with meat and hobby farming as secondary purposes. The majority of the participants in our questionnaire were meat-producing sheep farmers, reflecting, therefore, the overall structure of the Belgian small ruminant sector.

The questionnaire revealed important shortcomings in the management of breeding males and highlighted certain differences in relation to disease prevention strategies between small ruminant farms in Flanders and Wallonia. Flemish farmers appear to adhere rigorously to best management practices, particularly those concerning the acquisition and movement of breeding males. In Flanders, a remarkably high percentage of farmers—73.7%—are aware of the health status of the source farms when purchasing or borrowing breeding males, compared to 52.0% in Wallonia. Also, Flemish farmers seldom lend animals to other farms, with only 17.7% doing so, compared to 28.8% in Wallonia. The Flemish region borders the south of the Netherlands, which faced a large human Q fever outbreak between 2007 and 2009, attributed to shedding of *C. burnetii* in dairy goats (26, 27). This prompted the implementation of an extensive set of control measures in the Dutch small ruminant sector, aiming at reducing shedding and environmental contamination (28), and finally resulting in the end of this outbreak (26, 29). The more consistent implementation of best management practices by farmers in the Flemish region—harboring itself areas with intensive goat farming comparable to those in the southern Netherlands—may be attributed to its geographical proximity and the strong interconnections with the Dutch small ruminant sector. These close ties likely raised awareness of the importance of strict biosecurity protocols and health monitoring of incoming animals among the Flemish farmers, fostering a culture of increased risk perception and preventive action. Nevertheless, the questionnaire revealed that a considerable proportion of farmers do not vaccinate their breeding males when vaccinating against diseases, with this habit being present in both regions albeit more pronounced in Wallonia (55.8%) compared to Flanders (25.8%). Incomplete herd vaccination leads to reduced and destabilized herd immunity, facilitating disease introduction and persistent circulation of pathogens within the flock (9). In the context of controlling sexually transmitted diseases such as Q fever, the act of not vaccinating breeding males poses an exacerbated risk, as natural service is the dominant breeding practice in the Belgian small ruminant sector.

The observed shortcomings in breeding practices on Belgian small ruminant farms underline the importance of systematic screening of breeding males prior to (re)introduction as part of disease control. Encouragingly, a substantial proportion of farmers—up to 78.8% in Wallonia and 61.3% in Flanders—recognizes the potential utility of systematic pre-purchase screening tests for sexually transmitted diseases. The primary motivation cited—maintaining a disease-free status—indicates a strong willingness to apply such preventive screening. However, barriers such as the cost remain a major constraint, alongside the belief, particularly for Flemish farmers, that purchasing animals exclusively from certified farms reduces the need for additional screening. The latter could explain the slightly lower interest of the Flemish farmer in pre-purchase screening tests. On the other hand, with current testing methods, it is very difficult to fully certify a Q fever-free status. In addition, lacking knowledge regarding disease risks and available diagnostic options keep farmers reluctant. Therefore, educational outreach, economic incentives, and the development of affordable and reliable screening tests might collectively support the broader implementation of pre-introduction screening strategies in the small ruminant sector.

The second part of this study aimed at evaluating the diagnostic performance of two *C. burnetii*-specific CMI tests—the IFN-γ recall assay on whole blood and intradermal testing—in sheep and goats under field circumstances and comparing their diagnostic performance with that of real-time qPCR on genital swabs and ELISA antibody testing. As the control of Q fever in Belgium is legally regulated, with measures including mandatory vaccination at the owner’s expense following a positive PCR result, farm recruitment for participation in this field study was particularly challenging.

We showed that the diagnostic performance of ELISA and the IFN-γ test was similar. However, IFN-γ testing appeared to have slightly higher DSe compared to ELISA, when additionally considering PCR and intradermal tests results. On Farm 2, four animals tested positive by PCR at a timepoint when no other test data were available. As this suggests a high Q fever prevalence on this farm, the latter also showed the most discrepant IFN-γ results: 12 discrepant results I+/E− vs. only one discrepant result in the opposite direction (I−/E+). Furthermore, the only animal with a positive intradermal test (Farm 4) tested IFN-γ-positive at the same time point, while ELISA was negative.

Interestingly, both the ELISA and IFN-γ tests perfectly agreed in the correct diagnosis of the negatively categorized Farms 6 to 9, suggesting good performances for DSp in both tests, which was further confirmed by all diagnostic tests throughout the entire study. However, contradictory results were observed on Farm 10. On this farm, one buck consistently tested positive in the ELISA. It is plausible that this animal, who was purchased without knowing its historical background, had been vaccinated in the past. From the two other breeding bucks present on Farm 10, one seroconverted and became positive in the *C. burnetii* IFN-γ recall assay at the time of the second pre-breeding visit of year two, and another one tested positive in the IFN-γ test in the same last two sampling points. It cannot be excluded that these animals were infected with *C. burnetii* during the study or that antibody ELISA and IFN-γ positivity in these two animals might have risen following (multiple) application(s) of the intradermal test. When sensitization through vaccination or infection has occurred in the past (even up to many years ago), intradermal antigen injection can result in the rise of both antigen-specific humoral and cellular immune responses. This so-called “booster effect” is well described upon intradermal testing for Q fever in humans (30, 31) and for tuberculosis in cattle (32), and is also observed in skin test-negative animals (30). Due to late recruitment of the farm and the lack of the three pre-breeding visits in year 1, follow-up on year 2, including the same breeding bucks, was attempted. This resulted in one intradermal test application post-breeding during year one and one application during the year 2 pre-breeding period, hence allowing us to notice this possible interference of the intradermal test with the ELISA and IFN-γ tests.

In the five farms with a Q fever-positive status, the Q fever-positivity rate varied considerably, a variability that has also been observed previously in field settings (9). On Farms 1 to 4, evidence of a Q fever infection was found by at least one out of four considered test methods, confirming their positive status. However, on Farm 1, the positivity rate was quite low with only one IFN-γ positive on 15 samples. This farm could have been incorrectly classified as positive, or the low prevalence may not have been fully detected due to the small sample size for blood sampling. In the absence of *C. burnetii*-positive genital swabs, the status of Farm 5 could not be fully confirmed, as the animals were recently vaccinated and both the ELISA and IFN-γ recall assays could not clearly differentiate between recent vaccination and infection. Surprisingly, direct detection of the bacterium by means of real-time qPCR on genital swabs was successful only on Farms 2 and 4, yielding a positivity rate of barely 1% in all swabs collected on the Q fever-positive farms. As a consequence, comparisons between real-time qPCR and the CMI tests were compromised. While detailed knowledge on *C. burnetii* excretion patterns in small ruminant males is lacking, previous studies showed that the number of female shedders in vaginal fluids is the highest during the first days following abortions or normal lambing (4, 5, 16). Also, vaginal excretion tends to be longer (up to 7 weeks post abortion) and at a higher level in aborting females compared to their non-aborting counterparts (16). During the study period, no abortions were observed. Next to that, Q fever vaccination was already in place on two out of five positive farms, lowering the likelihood of direct detection and at least partially explaining the low real-time qPCR positivity rate and its decrease over time (1% pre-breeding vs. 0.2% post-breeding). Overall, although plausible that active shedding was minimal to no longer present in most farms during the study period, the sensibility/suitability of qPCR for systematic screening purposes remains questionable, especially in the absence of clinical symptoms.

While intradermal testing has been identified as an effective method for detecting *C. burnetii*-immune animals in cattle herds (20, 21), our study observed a low positivity rate (1/44 tests). Although the readings of the intradermal tests were re-evaluated by the investigating scientists through photographs in the majority of cases, we cannot completely rule out the possibility of interpretation errors by the reporting farmers. As such, results derived from intradermal testing were particularly used to interpret and confirm the ELISA and IFN-γ test results. Notably, none of the 27 skin-tested males tested positive by real-time qPCR. In this group, both antibody and IFN-γ positivity rates were low, at 33.3% (9/27 animals). It is likely that the prevalence of *C. burnetii* infection among these males was insufficient to yield the number of positive results required for robust test evaluation.

Our results demonstrated that the IFN-γ recall assay can be successfully performed using whole blood directly, skipping time- and labor-consuming PBMC isolation (18). Furthermore, we showed that antigen stimulation time can be reduced up to 24 h instead of 48 h without compromising the discriminatory ability of the test. Those adaptations improved the test’s accessibility for application in field settings, although it remains a test that requires important logistic operations. A promising solution would be to perform antigen stimulation directly on whole blood while minimizing incubation time, comparable to the Q-detect™ CMI test (Innatoss Laboratories, Oss, Netherlands) for Q fever diagnostics in humans (33). While the IFN-γ test remains logistically more challenging and more time consuming than the antibody ELISA, we estimate the former to be approximately one-third cheaper in our laboratory settings. The difference in cost can be explained by the use of an in-house produced antigen for *in-vitro* simulation in the IFN-γ test, and by the higher equipment costs associated with the antibody ELISA due to its more complex assay protocol. With an overall DSe of 0.80 and DSp 0.94, diagnostic performance of the IFN-γ test under field conditions was comparable to that of the antibody ELISA (DSe of 0.74 of and DSp of 0.96). However, when excluding vaccinated animals, the IFN-γ recall assay appeared to be more sensitive than the ELISA. These observations can be explained by the fact that the two tests rely on a different part of the immune system. The IFN-γ recall assay targets the cellular immunity, measuring the presence of antigen-specific IFN-γ-producing cells, while the ELISA addresses the humoral immunity by detecting antibodies. Upon exposure, the production of *C. burnetii*-specific IgG antibodies targeted in most ELISA essays requires several weeks (18, 19, 34). In contrast, antigen-specific IFN-γ responses are detectable within days after *C. burnetii* infection (18). Hence, infected animals can be detected faster with the IFN-γ test than with the ELISA. In addition, *C. burnetii* infection provokes an elaborate cell-mediated immune response, rather than strong humoral responses, due to the intracellular lifestyle of the bacterium (18), further favoring detection with the IFN-γ test when vaccination is not applied. Vice versa, vaccination with the *C. burnetii* vaccine Coxevac®—an inactivated, non-adjuvanted vaccine—elicits a predominant humoral immune response strongly boosted upon infection (18, 19). Vaccination also boosts immune responses to a previous Q fever infection, leading to long-lasting seroconversion (35) and explaining the high ELISA signals observed on the vaccinating Q fever-positive farms. Therefore, the potential of the IFN-γ recall assay to detect previously infected animals is particularly valuable in field settings where Q fever vaccination is not implemented or in animals that have not been vaccinated against the disease. When vaccination is applied, knowledge of the vaccination status and the time of administration is essential for accurate interpretation of both the antibody ELISA and IFN-γ test, as vaccination influences—in different extent—test results.

In conclusion, the questionnaire distributed to Belgian sheep and goat farmers highlighted the relevance of screening for Q fever and other sexually transmitted diseases when purchasing breeding males. This is particularly important given the lack of information about both the health status of the source farm and the vaccination history of the acquired animals. The latter information is crucial, not only in the context of good biosecurity practices but also for reliable interpretation of test results. While such screenings are not commonly performed in practice, most farmers consider systematic testing for sexually transmitted diseases (e.g., leptospirosis, chlamydiosis, Q fever) at the time of purchase to be useful. A comparison of two *C. burnetii*-specific CMI assays with established diagnostic methods under field conditions identified the IFN-γ recall assay as a promising tool. Its sensitivity and specificity were comparable to those of antibody ELISA, with notably higher sensitivity in unvaccinated contexts.

## Data Availability

The raw data supporting the conclusions of this article will be made available by the authors, without undue reservation.
