# Supplementary material for: Pre-purchase screening for Coxiella burnetii in small ruminants: farm acceptance and field evaluation identify the ex-vivo interferon-γ assay as a promising tool
Source: Front Vet Sci. 2025 Dec 4;12:1708200. doi: 10.3389/fvets.2025.1708200 (PMC12711480; doi:10.3389/fvets.2025.1708200)
Supplement: Supplementary file 1 [file Data_Sheet_1.PDF]

Questionnaire (French)

Introduction

Chers éleveurs d'ovins et de caprins,

Comme vous savez, l'achat d'animaux est un facteur de risque important pour l'introduction et la propagation de maladies contagieuses dans votre exploitation. Cependant, les risques entraînés par l'achat d'animaux reproducteurs mâles, en particulier, ont été peu étudiés jusqu'à présent. Il est probable que les béliers et les boucs reproducteurs achetés, dont le statut infectieux est inconnu, présentent un risque élevé d'introduction et de propagation de maladies sexuellement transmissibles (telles que la leptospirose, la chlamydiose et la fièvre Q), car ils entrent en contact avec de nombreuses femelles pendant la saison de reproduction. Contrôler ces animaux avant de les autoriser à entrer dans le troupeau serait donc une bonne stratégie pour réduire le risque d'infection. En outre, ils peuvent être d'excellentes sentinelles à cause de leurs nombreux contacts. Par conséquent, l'échantillonnage régulier des mâles reproducteurs peut également être un bon outil pour surveiller la santé du troupeau.

Par le biais de l'enquête ci-dessous, Sciensano, la DGZ (côté flamand) et l'ARSIA (côté wallon) veulent avoir un aperçu de la gestion des mâles reproducteurs dans les exploitations ovines et caprines en Belgique, et vérifier la perception d'un test d'achat pour ces animaux afin de réduire la (ré)émergence des maladies sexuellement transmissibles dans les exploitations. Cette enquête est liée à un nouveau projet de recherche financé par le Fonds Sanitaire. Le but de ce projet? Évaluer l'utilisation d'un test de dépistage pour la fièvre Q chez les béliers et les boucs reproducteurs. Pour cela nous recherchons des exploitations ovines et caprines avec et sans problèmes de fièvre Q où nous pouvons suivre l'état infectieux des mâles reproducteurs et d'une partie des femelles pendant un an au moyen de différents types de tests. Êtes-vous intéressé(e) par cette recherche? Remplissez vos coordonnées à la fin de l'enquête!

L'enquête prend environ 10 minutes à remplir. Nous vous remercions d'avance pour votre participation!

1 Structure et gestion de l'exploitation

Question 1/ Dans quelle province votre exploitation est-elle située?

- □ Hainaut
- □ Liège
- □ Luxembourg
- □ Namur
- □ Brabant Wallon

Question 2/ Quel est votre code postal?

Question 3/ Quelle(s) activité(s) a/ont lieu dans votre exploitation?

□ La production de lait de brebis
□ La production de lait de chèvre
□ La production et l’engraissement d’agneaux
□ La production et l’engraissement de chevreaux
□ L’élevage d’ ovins destinés à la reproduction
□ L’élevage de caprins destinés à la reproduction

Question 4/ Quel est le nombre total d’ovins et/ou de caprins présents actuellement dans votre exploitation?

Question 5/ Quel est le nombre de femelles en âge de se reproduire présentes actuellement dans votre exploitation?

Question 6/ Les chaleurs sont-elles induites en dehors de la saison de reproduction?

□ Non
□ Oui

Question 7/ Précisez la méthode d’induction des chaleurs (par exemple à l’aide d’éponges,…):

Question 8/ Les chaleurs sont-elles regroupées pendant la période de reproduction?

□ Non
□ Oui

Question 9/ Précisez la méthode de synchronisation des chaleurs (par exemple à l’aide du bélier/bouc, éponges,…):

Question 10/ Comment les brebis/chèvres sont-elles saillies?

□ Exclusivement par le bélier/bouc (saillie naturelle)
□ Par le bélier/bouc (saillie naturelle) et par insémination artificielle
□ Exclusivement par insémination artificielle

Question 11/ Au cours de quel(s) mois les saillies ont-elles lieu?

□ Janvier
□ Février
□ Mars
□ Avril
□ Mai
□ Juin
□ Juillet
□ Août
□ Septembre

□ Octobre
□ Novembre
□ Décembre

Question 12/ Élevez-vous, en plus des ovins et/ou caprins, d'autres animaux? Si oui, lesquels?

□ Non
□ Oui, d'autres petits ruminants, y compris les cervidés
□ Oui, des bovins
□ Oui, des porcs
□ Oui, de la volaille
□ Oui, des chevaux
□ Oui, d'autres animaux que ceux listés ci-dessus

2 Le bélier/bouc

Question 13/ Combien de mâles reproducteurs votre exploitation compte-t-elle?

Question 14/ Après combien d'années les mâles reproducteurs sont-ils remplacés?

Question 15/ Les mâles reproducteurs qui vous utilisez sur votre exploitation sont

□ achetés
□ empruntés
□ nés dans votre exploitation

Question 16 à 20: ne répondez à ces questions que si vous achetez et/ou empruntez des mâles reproducteurs

Question 16/ Précisez le nombre des élevages d'origine:

□ *1*
□ *2*
□ *3*
□ *4*
□ *5*
□ *>5*

Question 17/ Êtes-vous au courant du statut sanitaire de tous les élevages d'origine?

□ *Non*
□ *Oui*

Question 18/ Connaissez-vous le statut vaccinal de tous les animaux achetés et/ou empruntés?

□ *Non*
□ *Oui*

Question 19/ Mettez-vous les animaux achetés et/ou empruntés en quarantaine après leur arrivée? Si oui, combien de temps?

□ *Non*
□ *Oui, 1 semaine*
□ *Oui, 2 semaines*
□ *Oui, 3 semaines*
□ *Oui, 4 semaines*
□ *Oui, 5 semaines*
□ *Oui, plus de 5 semaines*
□ *Oui, autre que listé ci*-dessus

Question 20/ Les animaux sont-ils dépistés pour certaines maladies contagieuses avant leur introduction dans le troupeau?

□ *Non*
□ *Oui*

Question 21/ Précisez pour quelle(s) maladie(s) les animaux achetés/empruntés sont dépistes avant leur introduction dans le troupeau:

Question 22/ Prêtez-vous également des mâles reproducteurs à d'autres élevages?

□ *Non*
□ *Oui*

<u>Question 23 à 26: ne répondez à ces questions que si vous prêtez des mâles reproducteurs à d'autres élevages</u>

Question 23/ À combien d'élevages prêtez-vous des animaux?

□ 1
□ 2
□ *3*
□ *4*
□ *5*
□ *>5*

Question 24/ Êtes-vous au courant du statut sanitaire de tous les élevages auxquels des animaux sont prêtés?

□ *Non*
□ *Oui*

Question 25/ Mettez-vous les animaux prêtés en quarantaine après leur retour? Si oui, combien de temps?

□ *Non*
□ *Oui, 1 semaine*
□ *Oui, 2 semaines*
□ *Oui, 3 semaines*
□ *Oui, 4 semaines*
□ *Oui, 5 semaines*
□ *Oui, plus de 5 semaines*
□ *Oui, autre que listé ci*-dessus

Question 26/ Les animaux sont-ils dépistés pour certaines maladies contagieuses avant leur réintroduction dans le troupeau?

□ *Non*
□ *Oui*
Précisez quelles maladie(s):
Question 27/ Précisez pour quelle(s) maladie(s) les animaux prêtés sont dépistes avant leur réintroduction dans le troupeau:

Question 28/ De quelle manière les mâles reproducteurs sont-ils employés durant la lutte?

□ Un ou plusieurs béliers/boucs sont placés temporairement dans un lot de brebis/chèvres (qui sont en chaleur ou pas encore) et saillissent librement

□ Le(s) bélier(s)/bouc(s) sailli(ssen)t 'en main' (les femelles en chaleur sont amenées individuellement au mâle)

□ Un ou plusieurs mâles restent toute l'année chez les femelles et sailli(ssen)t librement

□ Autre…

Question 29/ Précisez, si applicable, le nombre de mâles reproducteurs par lot de femelles à saillir et combien de temps le(s) bélier(s)/bouc(s) reste(nt) chez les femelles (par exemple 1 bélier par 30 femelles pendant 3 semaines):

Question 30/ Les mâles reproducteurs sont-ils échangés entre différents lots de femelles dans l'exploitation durant la période de lutte?

□ Non
□ Oui

Question 31/ Combien de femelles un bélier/bouc saillit-il pendant la période de lutte?

Question 32/ Combien de femelles sont saillies au total pendant la période de lutte?

3 La santé animale

Question 33/ Des problèmes de santé animale ont-ils été observés dans votre exploitation au cours de l'année passée, en particulier des avortements, des mises-bas prématurées et/ou des nouveaux nés chétifs?

- □ Non
- □ Oui

Question 34/ Au cours de l'année passée, une infection par la fièvre Q a-t-elle été diagnostiquée dans votre exploitation via le monitoring de lait de tank ou le protocole avortement?

- □ Non
- □ Oui

Question 35/ Vaccinez-vous contre la fièvre Q en ce moment?

- □ Non
- □ Oui, systématiquement (indépendamment du résultat du monitoring de lait de tank ou du protocole avortement)
- □ Oui, mais pas systématiquement (seulement après un résultat non-conforme du monitoring de lait de tank ou du protocole avortement)

Question 36/ Si vous vaccinez contre des maladies, les mâles reproducteurs sont-ils aussi vaccinés?

- □ Non
- □ Oui

4 La perception d'un test des maladies sexuellement transmissibles à l'achat des mâles reproducteurs

Question 37/ Pensez-vous qu'il est judicieux de dépister systématiquement les mâles reproducteurs à l'achat pour des maladies sexuellement transmissibles (par exemple la leptospirose, la chlamydiose, la fièvre Q)?

- □ Non
- □ Oui

Question 38/ Quel est pour vous la motivation principale d'utiliser un test d'achat pour des maladies sexuellement transmissibles?

- □ Obtenir plus d'informations sur le statut sanitaire des animaux achetés
- □ Améliorer le statut sanitaire de l'exploitation en diminuant la pression infectieuse
- □ Maintenir un statut "indemne de maladie"
- □ Autre…

Question 39/ Quel est pour vous la motivation principale de ne pas utiliser un test d'achat pour des maladies sexuellement transmissibles?

□ Le coût
□ L'incertitude sur la poursuite/continuation de la vente
□ Autre…

Question 40/ Combien êtes-vous prêt(e) à payer pour un test d'achat pour des maladies sexuellement transmissibles?

Question 41/ Combien de temps êtes-vous prêt(e) à attendre entre le prélèvement et la réception du résultat?

Question 42/ Sciensano, la DGZ (côté flamand) et l'ARSIA (côté wallon) sont à la recherche d'exploitations ovines et caprines qui souhaiteraient participer à une étude sur le terrain afin d'évaluer l'utilisation d'un test de dépistage pour la fièvre Q chez les béliers et les boucs reproducteurs. Êtes-vous intéressé(e) par cette recherche?

□ Non
□ Oui

Question 43/ Vos coordonnées (nom, adresse e-mail et/ou numéro de téléphone):

Questionnaire (Dutch)

Inleiding

Beste schapen- en geitenhouders,

Zoals jullie weten, is de aankoop van dieren een belangrijke risicofactor voor de insleep en spreiding van besmettelijke ziekten op uw bedrijf. De risico's die de aankoop van mannelijke fokdieren in het bijzonder met zich meebrengt, heeft men tot op heden echter weinig onderzocht. Het is aannemelijk dat aangekochte dekrammen en –bokken met een ongekende infectiestatus een groot risico vormen voor insleep en spreiding van seksueel overdraagbare ziekten (zoals bijvoorbeeld leptospirose, chlamydiose en Q-koorts), daar zij tijdens het dekseizoen met talrijke vrouwtjes in contact komen. Deze dieren controleren voordat ze in de kudde worden toegelaten zou dus een goede strategie zijn om de kans op infectie te beperken. Bovendien kunnen ze door hun vele contacten fungeren als uitstekende verklikkerdieren. Daarom kunnen regelmatige staalnames bij de mannetjes ook een goede tool vormen om de gezondheid van de kudde te bewaken.

Via onderstaande enquête willen Sciensano, DGZ (aan Vlaamse zijde) en ARSIA (aan Waalse zijde) inzicht krijgen in het beheer van de mannelijke fokdieren op schapen- en geitenbedrijven in België, en de perceptie nagaan van een aankooptest voor deze dieren om het (opnieuw) voorkomen van seksueel overdraagbare ziekten op bedrijven te beperken. Deze enquête is gelinkt aan een nieuw onderzoeksproject dat wordt gefinancierd door het Sanitair Fonds. Het doel van dit project? Het gebruik evalueren van een screeningstest voor Q-koorts bij dekrammen en –bokken. Daarvoor zijn we op zoek naar schapen- en geitenbedrijven met en zonder Q-koorts problematiek waar we de infectiestatus van de mannelijke fokdieren en een deel van de vrouwtjes gedurende een jaar kunnen opvolgen aan de hand van verschillende types testen. Heeft u interesse in dit onderzoek? Vul dan uw contactgegevens in op het einde van de enquête!

Het invullen van de enquête neemt ongeveer 10 minuten in beslag. Alvast heel erg bedankt voor uw deelname!

1 bedrijfsstructuur en bedrijfsvoering

Vraag 1/ In welke provincie is uw bedrijf gelegen?

- □ Antwerpen
- □ Limburg
- □ Oost-Vlaanderen
- □ Vlaams-Brabant
- □ West-Vlaanderen

Vraag 2/ Wat is uw postcode?

Vraag 3/ Welke activiteit(en) vindt/vinden plaats op uw bedrijf?

□ Productie van schapenmelk
□ Productie van geitenmelk
□ Het kweken en afmesten van schapenlammeren
□ Het kweken en afmesten van geitenlammeren
□ Het kweken van schapen bestemd voor de fok
□ Het kweken van geiten bestemd voor de fok

Vraag 4/ Wat is op vandaag het totaal aantal schapen en/of geiten aanwezig op uw bedrijf?

Vraag 5/ Wat is op vandaag het aantal vrouwelijke dieren van vruchtbare leeftijd aanwezig op uw bedrijf?

Vraag 6/ Worden de dieren in bronst gebracht buiten het bronstseizoen?

□ Nee
□ Ja

Vraag 7/ Specifieer de manier van bronstinductie (bijvoorbeeld met behulp van sponzen, …):

Vraag 8/ Worden de bronsten gegroepeerd tijdens de bronstperiode?

□ Nee
□ Ja

Vraag 9/ *Specifieer de manier van bronstsynchronisatie (bijvoorbeeld met behulp van de ram/bok, sponzen, …):*

Vraag 10/ Hoe worden de ooien/geiten gedekt?

□ Uitsluitend door de ram/bok (natuurlijke dekking)
□ Door de ram/bok (natuurlijke dekking) en via kunstmatige inseminatie
□ Uitsluitend via kunstmatige inseminatie

Vraag 11/ In welke maand(en) vinden de dekkingen plaats?

□ Januari
□ Februari
□ Maart
□ April
□ Mei
□ Juni
□ Juli
□ Augustus
□ September
□ Oktober
□ November
□ December

Vraag 12/ Houdt u, naast schapen en/of geiten, ook andere dieren? Zo ja, welke?

□ Nee
□ Ja, andere kleine herkauwers waaronder hertachtigen
□ Ja, runderen
□ Ja, varkens
□ Ja, pluimvee
□ Ja, paarden
□ Ja, andere dan hierboven vermeld

2 De ram/bok

Vraag 13/ Hoeveel mannelijke fokdieren zijn er op vandaag aanwezig op uw bedrijf?

Vraag 14/ Na hoeveel jaar worden de mannelijke fokdieren gemiddeld vervangen?

Vraag 15/ De mannelijke fokdieren die u inzet op uw bedrijf zijn

□ aangekocht
□ geleend
□ geboren op uw bedrijf

Vraag 16 t.e.m. 20: enkel te beantwoorden indien u mannelijke fokdieren aankoopt en/of leent

Vraag 16/ Specifieer het aantal herkomstbedrijven:

□ *1*
□ *2*
□ *3*
□ *4*
□ *5*
□ *>5*

Vraag 17/ Bent u op de hoogte van de gezondheidsstatus van alle herkomstbedrijven?

□ *Nee*
□ *Ja*

Vraag 18/ Kent u de vaccinatiestatus van alle aangekochte en/of geleende dieren?

□ *Nee*
□ *Ja*

Vraag 19/ Plaatst u de aangekochte en/of geleende dieren na aankomst in quarantaine? Zo ja, hoelang?

□ *Nee*

□ *Ja, 1 week*
□ *Ja, 2 weken*
□ *Ja, 3 weken*
□ *Ja, 4 weken*
□ *Ja, 5 weken*
□ *Ja, meer dan 5 weken*
□ *Ja, anders dan hierboven vermeld*

Vraag 20/ Worden de dieren gescreend op bepaalde besmettelijke ziekten vooraleer ze in de kudde geïntroduceerd worden?

□ *Nee*
□ *Ja*

Vraag 21/ Specifieer op welke ziekte(s) de aangekochte/geleende dieren gescreend worden vóór introductie in de kudde:

Vraag 22/ Leent u zelf mannelijke fokdieren uit aan andere bedrijven?

□ Nee
□ Ja

<u>Vraag 23 t.e.m. 26: enkel te beantwoorden indien u mannelijke fokdieren uitleent</u>

Vraag 23/ Aan hoeveel bedrijven leent u dieren uit?

□ 1
□ 2
□ *3*
□ *4*
□ *5*
□ *>5*

Vraag 24/ Bent u op de hoogte van de gezondheidsstatus van alle bedrijven waaraan dieren worden uitgeleend?

□ *Nee*
□ *Ja*

Vraag 25/ Plaatst u de uitgeleende dieren na terugkomst in quarantaine? Zo ja, hoelang?

□ *Nee*
□ *Ja, 1 week*
□ *Ja, 2 weken*
□ *Ja, 3 weken*
□ *Ja, 4 weken*
□ *Ja, 5 weken*
□ *Ja, meer dan 5 weken*

□ *Ja, anders dan hierboven vermeld*

Vraag 26/ Worden de dieren gescreend op bepaalde besmettelijke ziekten vooraleer ze terug in de eigen kudde geïntroduceerd worden?

□ *Nee*
□ *Ja*

Vraag 27/ Specifieer op welke ziekte(s) de uitgeleende dieren gescreend worden vóór herintroductie in de kudde:

Vraag 28/ Op welke manier(en) worden de mannelijke fokdieren ingezet tijdens het dekken?

□ Eén of meerdere rammen/bokken worden tijdelijk geplaatst in een groep ooien/geiten (die al dan niet reeds bronstig zijn) en dekken vrij

□ De ram(men)/bok(ken) dekt/dekken uit de hand (bronstige ooien/geiten worden één voor één bij de ram/bok gebracht)

□ Eén of meerdere rammen/bokken verblijven het hele jaar door bij de vrouwelijke dieren en dekken vrij

□ Anders…

Vraag 29/ Specifieer indien van toepassing het aantal mannelijke fokdieren per groep te dekken vrouwtjes en hoelang de ram(men)/bok(ken) bij de vrouwelijke dieren verblijft/verblijven (bijvoorbeeld: 1 mannetje per 30 vrouwtjes gedurende 3 weken):

Vraag 30/ Worden de mannelijke fokdieren tijdens de dekperiode uitgewisseld tussen verschillende groepen vrouwtjes binnen het bedrijf?

□ Nee
□ Ja

Vraag 31/ Hoeveel vrouwelijke dieren dekt één ram/bok gedurende de dekperiode?

Vraag 32/ Hoeveel vrouwelijke dieren worden er in totaal gedekt gedurende de dekperiode?

3 Diergezondheid

Vraag 33/ Zijn er in het voorbije jaar op uw bedrijf gezondheidsproblemen geweest, met name verwerpingen, vroeggeboortes en/of zwakgeboren lammeren?

□ Nee
□ Ja

Vraag 34/ Werd er in het voorbije jaar een Q-koorts infectie op uw bedrijf vastgesteld via het tankmelkonderzoek of het abortusprotocol?

□ Nee

□ Ja

Vraag 35/ Wordt er momenteel op uw bedrijf gevaccineerd tegen Q-koorts?

□ Nee

□ Ja, dit gebeurt systematisch (onafhankelijk van het resultaat van het tankmelkonderzoek of abortusprotocol)

□ Ja, maar dit gebeurt niet systematisch (enkel na een niet-conform resultaat van het tankmelkonderzoek of abortusprotocol)

Vraag 36/ Indien er gevaccineerd wordt tegen ziektes, worden de mannelijke fokdieren steeds mee gevaccineerd?

□ Nee

□ Ja

4 Perceptie van een test op seksueel overdraagbare ziekten bij de aankoop van mannelijke fokdieren

Vraag 37/ Is het volgens u zinvol om mannelijke fokdieren bij aankoop systematisch te screenen op seksueel overdraagbare ziektes (bijvoorbeeld leptospirose, chlamydiose, Q-koorts)?

□ Nee

□ Ja

Vraag 38/ Wat is voor u de voornaamste beweegreden om gebruik te maken van een aankooptest voor seksueel overdraagbare ziektes?

□ Meer informatie verkrijgen over de gezondheidsstatus van aangekochte dieren

□ Verbeteren van de gezondheidsstatus van het bedrijf door het verlagen van de infectiedruk

□ Behouden van een ziektevrije bedrijfsstatus

□ Andere…

Vraag 39/ Wat is voor u de voornaamste beweegreden om geen gebruik te maken van een aankooptest voor seksueel overdraagbare ziektes?

□ De kostprijs

□ Onzekerheid over het doorgaan van de verkoop

□ Andere…

Vraag 40/ Hoeveel bent u bereid te betalen voor een aankooptest voor seksueel overdraagbare ziektes?

Vraag 41/ Hoelang bent u bereid te wachten tussen de afname van de aankooptest en de ontvangst van het testresultaat?

Vraag 42/ Sciensano, DGZ (aan Vlaamse zijde) en ARSIA (aan Waalse zijde) zijn op zoek naar schapen-en geitenbedrijven die willen deelnemen aan een veldonderzoek om het gebruik van een

screeningstest voor Q-koorts bij dekrammen en -bokken te evalueren. Heeft u interesse in dit onderzoek?

□ Nee

□ Ja

Vraag 43/ Uw contactgegevens (naam, emailadres en/of telefoonnummer):
