# Supplementary material for: Pre-purchase screening for Coxiella burnetii in small ruminants: farm acceptance and field evaluation identify the ex-vivo interferon-γ assay as a promising tool
Source: Front Vet Sci. 2025 Dec 4;12:1708200. doi: 10.3389/fvets.2025.1708200 (PMC12711480; doi:10.3389/fvets.2025.1708200)
Supplement: Supplementary file 2 [file Table_1.DOCX]

Supplementary Material

Supplementary Figure 1: General characteristics of the participants of the questionnaire.


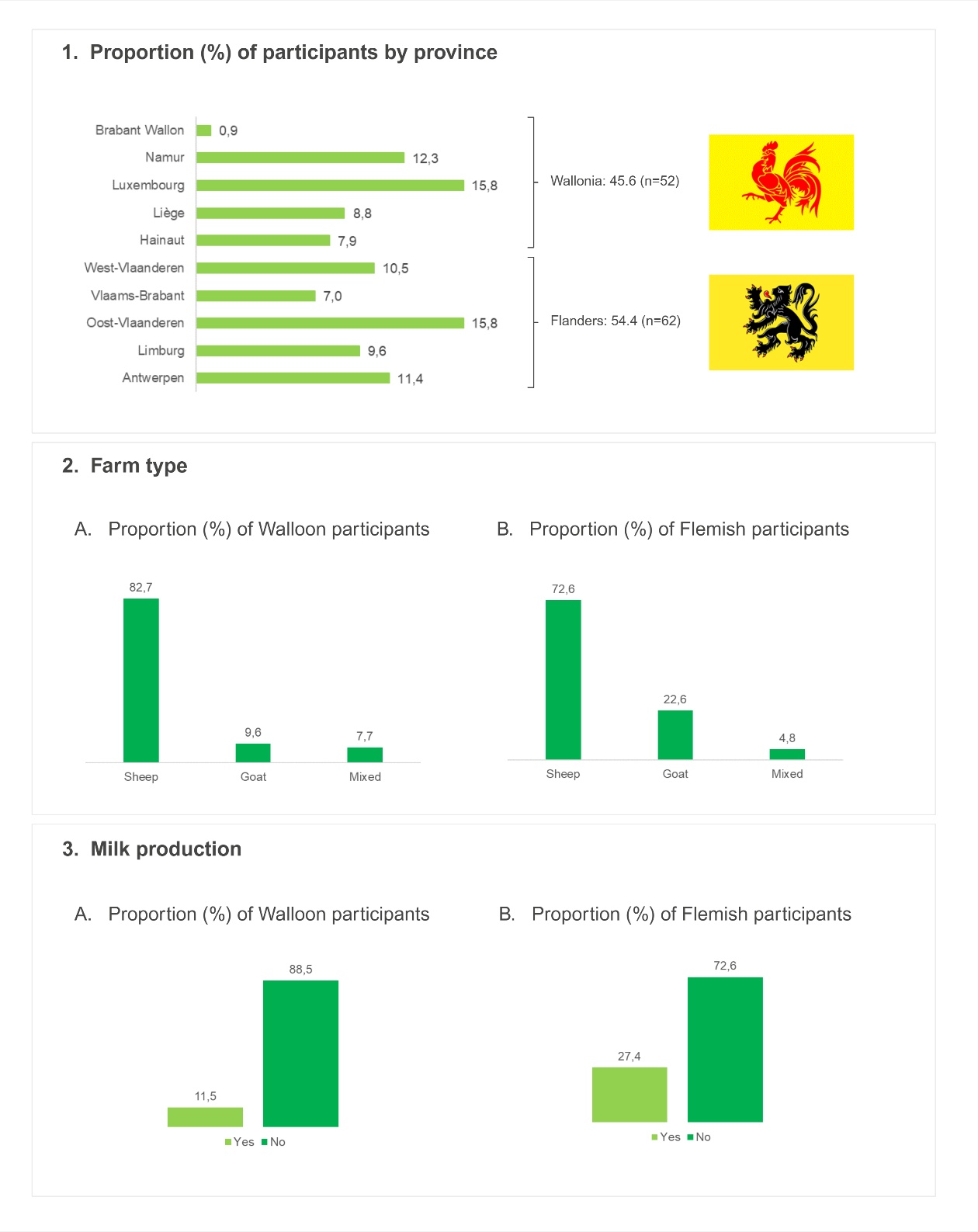


Supplementary Script 1: JAGS code used for evaluating diagnostic test characteristics of ELISA and IFNγ recall assay from sampling results in 10 sheep/goat farms.

model{

#=== LIKELIHOOD ===#

for (i in 1:10){ # 10 herds

obs[i, 1:4] ~ dmulti(p[i, 1:4], totals[i])

p[i, 1] <- prev[i]*Se_ELISA*Se_IFNg24 + (1-prev[i])*(1-Sp_ELISA)*(1-Sp_IFNg24) ## Pos/Pos

p[i, 2] <- prev[i]*Se_ELISA*(1-Se_IFNg24) + (1-prev[i])*(1-Sp_ELISA)*Sp_IFNg24 ## Pos/Neg

p[i, 3] <- prev[i]*(1-Se_ELISA)*Se_IFNg24 + (1-prev[i])*Sp_ELISA*(1-Sp_IFNg24) ## Neg/Pos

p[i, 4] <- prev[i]*(1-Se_ELISA)*(1-Se_IFNg24) + (1-prev[i])*Sp_ELISA*Sp_IFNg24 ## Neg/Neg

prev[i] ~ dbeta(alpha, beta)

}

#=== PRIOR ===#

alpha <- mu*psi ## a parameter for the hierarchical beta distribution

beta <- psi*(1-mu) ## b parameter for the hierarchical beta distribution

mu ~ dbeta(1, 1) T(0.0001,0.9999) ## Mean within-herd prevalence distribution in the source population

psi ~ dgamma(0.01, 0.01) T(0.0001,) ## Spread of the within-herd prevalence distribution in the source population

Se_ELISA ~ dbeta(1, 1) ## Prior for Se of ELISA

Sp_ELISA ~ dbeta(1, 1) ## Prior for Sp of ELISA

Se_IFNg24 ~ dbeta(1, 1) ## Prior for Se of IFNg24

Sp_IFNg24 ~ dbeta(1, 1) ## Prior for Sp of IFNg24

}

**Supplementary Table 1: Test results data used in the JAGS model.**

|  | ELISA / IFNγ | | | |
| --- | --- | --- | --- | --- |
| Farm | Pos/Pos | Pos/Neg | Neg/Pos | Neg/Neg |
| 1 | 0 | 0 | 1 | 15 |
| 2 | 24 | 1 | 12 | 5 |
| 3 | 3 | 2 | 6 | 38 |
| 4 | 29 | 4 | 4 | 9 |
| 5 | 14 | 7 | 5 | 14 |
| 6 | 0 | 0 | 0 | 9 |
| 7 | 0 | 0 | 0 | 6 |
| 8 | 0 | 0 | 0 | 18 |
| 9 | 0 | 0 | 0 | 3 |
| 10 | 2 | 6 | 2 | 8 |
